# Effect of Entomopathogenic Fungi, *Beauveria bassiana* (Cordycipitaceae), on the Bark Beetle, *Ips typographus* (L.), under Field Conditions

**DOI:** 10.3390/insects13100885

**Published:** 2022-09-29

**Authors:** Ciprian George Fora, Nicușor Boja, Mihaela Moatăr, Ferenc Tóth, Adalbert Balog

**Affiliations:** 1 Department of Forestry, Faculty of Horticulture and Forestry, Banat’s University of Agricultural Sciences and Veterinary Medicine “King Michael I of Romania” from Timișoara, Calea Aradului 119, 300645 Timișoara, Romania; 2 Research Institute of Organic Agriculture, Miklós tér 1., 1033 Budapest, Hungary; 3 Department of Horticulture, Faculty of Technical and Human Sciences, Sapientia Hungarian University of Transylvania, Aleea Sighișoarei 1C, Corunca, 540485 Târgu Mureș, Romania

**Keywords:** attack progression, biological control, fungal treatment, synthetic insecticide, climate change, mortality rate

## Abstract

**Simple Summary:**

The effect of *B. bassiana* fungal treatment on spruce bark beetle, *Ips typographus* (L.), was tested under field conditions. Altogether, it was detected that the effect of *B. bassiana* preparations on bark beetle infestations in terms of possible environmental effects is minimal compared to the severe damage and hence yield loss caused by this insect species worldwide. Even if the relatively low effects on I. typographus stages, including adults, larvae, pupae and even next-generation young adults, were reported, it remains clear that the present novel approach using a natural biological control agent rather than toxic synthetic insecticides is timely and may be potentially beneficial to the forestry industry as a rational control approach to combat this pest.

**Abstract:**

The spruce bark beetle, *Ips typographus* (L.), attack progression (adult and larval galleries) and parental and offspring mortality rate were assessed in managed forests of the Poiana Ruscă and Bihor Mountains, along with Western Romanian Carpathians using fungal (*Beauveria bassiana*) treatments. The results show that the effect of *B. bassiana* on adult (maternal) gallery length was similar to the untreated variant and was less effective than the synthetic insecticide lambda-cyhalothrin applied at a dose of 50 g/L. Additionally, its effect on the mean larval gallery number per maternal gallery was low. *B. bassiana* did not have a significant influence on the attack progression. Significant correlations between log diameter and *I. typographus* attack progression were detected; such differences were higher than the effect of any treatment. Altogether, abiotic (low humidity, high temperature) and biotic factors (log diameter) influenced the bark beetles’ attack progression and reduced the entomopathogenic fungal effects. Considering the efficacy of the *B. bassiana* treatment on logs infested with *I. typographus*, the results showed that parents and offspring were infected, but even if the fungal treatment was applied in high concentrations, the mortality rate remained relatively low. Further research is necessary to test if different *B. bassiana* strains and their commercially recommended concentrations might be more effective under dry and warm climate conditions, respectively.

## 1. Introduction

Forests are one of the planet’s most valuable resources, being constantly under abiotic, biotic and anthropogenic pressure. One of the factors significantly influencing forest dynamics is drought, which results in reduced tree growth and accelerated drying. In Europe, the area of forest affected by drought and drying exceeded 500,000 ha between 1987 and 2016 [1]. Together with drought, the increase in multiannual mean temperature has also had a significant an impact on forests. The vegetation and the photosynthesis period have duly increased, as well as the intensity of respiration. As a consequence, the formation of buds and leaves and their maturation have been disturbed [2]. 

A result of such obvious climate change is the periodic occurrence of storms that affect deciduous stands and, especially, coniferous forests, spruce trees being highly susceptible to climate-induced destruction and disease in Europe [3]. The frequency of major storms is continuing to intensify year by year and is likely to increase in the coming decades. Thus, while at the beginning of the century, storms were less intense and rare, as we approach the present day, storms are becoming more powerful and more frequent, and the damage caused amounts to millions of cubic meters of forest on hundreds of thousands of hectares. In Europe, an average of some 128,000 ha of forest are affected by storms each year. In addition to storms, fires have a major impact on forests. Thus, in Europe as a whole, around 142,000 ha of forest are affected by fires every year [3]. 

Prolonged droughts, rising temperatures, severe storms and fires are factors that influence forest dynamics differentially from region to region and create favorable conditions for plant diseases and pests. One of the most damaging insect pest species under the above-mentioned abiotic conditions remains the eight-toothed spruce bark beetle, *Ips typographus* (L.) (Coleoptera: Scolytidae). The species is a secondary pest that can produce mass outbreaks under certain conditions and can even attack healthy standing spruce trees (e.g., Norway spruce, *Picea abies* L.) during such outbreaks. Several methods have been investigated to control outbreaks, ranging from felling and extracting attacked trees or peeling dry trees to using synthetic pheromones to capture adults or spraying logs with synthetic pyrethroids. Each of these methods has its drawbacks, which is why new, innovative, highly effective and environmentally friendly solutions are urgently required and need to be tested. The use of microbiological preparations, such as entomopathogenic fungi, *B. bassiana*, may prove to be a solution in forest management to limit bark beetle attacks.

*B. bassiana* is a naturally occurring pathogen of *I. typographus* [4]. Its pathogenicity to bark beetles has been demonstrated in a few studies [5,6,7]; however, its efficacy is known to decrease over time [8]. The effect of this fungus on bark beetles is still limited, and further research is needed to investigate the effects, especially including the ideal concentration and comparisons with commercial synthetic insecticides under the aforementioned abiotic conditions.

Therefore, in this study, we have attempted to answer some of the questions related to the effective use of *B. bassiana*-based products, the followings questions being posed: (1) How does the commercial product Metab (*B. bassiana* product) applied at different concentrations influence the length of maternal galleries and the number of larval/maternal galleries? (2) Does *B. bassiana* cause mortality in adults and offspring? (3) Does *B. bassiana* cause mortality in young adults in the next generation? (4) Is Metab more effective than a commercially used synthetic insecticide? In this light, the bark beetle attack development was assessed under fungal and synthetic insecticide treatments in managed forest plots and in buffer zones of protected areas.

## 2. Material and Methods

### 2.1. Data Collection

**Figure 1:**
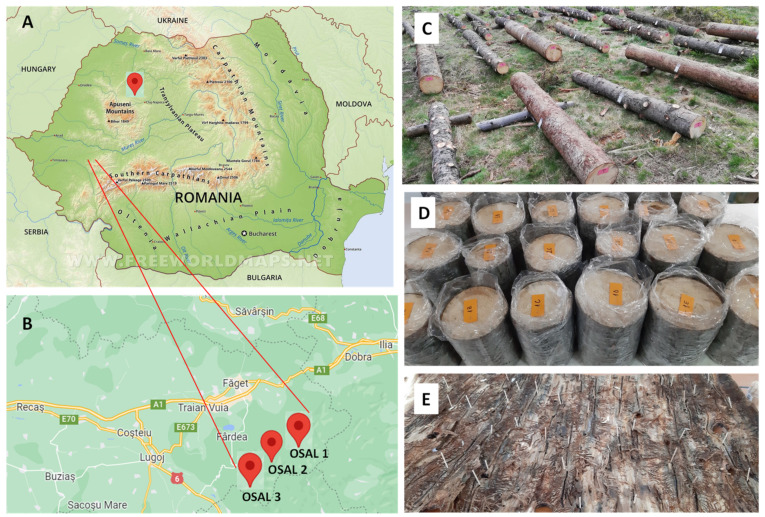
Experimental sites (**A**,**B**) (Google Map); plots with stems of branches of 1 m-long sections with diameters between 16 and 22 cm (**C**,**D**); galleries of *I. typographus* adults and larvae (**E**).

In order to answer the questions, three experiments were set up between 2019 and 2021 and conducted in three sites (OSAL 1, 2 and 3, where OSAL is the official name of the Forest Management Agency in Romania, Timiș, Romania) parallel in the vicinity of stands in which spruce represented 90% of trees and larch represented 10% of trees. The distance between the experimental sites was about 200 m. The sites were located outside the natural area, in the Poiana Ruscă Mountains of the Western Romanian Carpathians, at an altitude of 1100 m above sea level (a.s.l.) (45°40′45″ N; 22°17′16″ E). The forest stands had an average age of 110 years, a consistency of 0.9 and a production class of II (Figure 1A,B).

Trees were selected as wind felled trees during the winter period, which still had green crowns and no obvious spruce bark beetle infestations. They were cleaned of branches and sectioned every two meters, and the resulting logs were placed horizontally (so that they did not touch the ground) in Latin rectangle test platforms with six variations and four repetitions for each experiment. The logs were placed randomly and labelled. The diameter of the logs was then measured at the middle, and bark area was calculated for each one. Each sample was primed at the middle with the commercial pheromone Atratyp. The samples were sprayed as follows: variant 1 (V1) with water; variant 2 (V2) with Metab 75 mL/10 L water; variant 3 (V3) with Metab 105 mL/10 L water; variant 4 (V4) with Metab 135 mL/10 L water; variant 5 (V5) with Metab 165 mL/10 L water; variant 6 (V6) with lambda-cyhalothrin 50 g/L (Karate Zeon) 50 mL/10 L water. The spray solutions were prepared one day prior to the use of the microbiological product, according to the manufacturer’s instructions. To activate the microorganisms, the Metab control product and the Nutriaction product were always added in double the amount of the test product. Thus, for example, for (V2), 150 mL Nutriaction was added; for (V3), 210 mL Nutriaction was added; for (V4), 270 mL Nutriaction was added; for (V5), 330 mL Nutriaction was added. The Zeon Karate solution was prepared shortly before spraying. The amount of solution applied was 300 mL/m^2^ bark.

The first treatment application was made on 19 April 2019; the first set of samples for laboratory analysis was collected on 7 June 2019. Also on this latter date, the second treatment was applied, and, again, on 21 June 2019, the second set of samples was collected. These collections were made to avoid the flight of live insects from the experimental logs. The logs were transported to the laboratory two weeks after the last spraying period. There, the samples were peeled, and the following assessments were made: (1) The number of entries per bark sample subsequently expressed per 1 m^2^ of bark; (2) 10 attack systems were chosen from the middle of each bark sample, and the lengths of 10 maternal galleries per sample were measured; (3) existing larval galleries were counted in the measured maternal galleries (Figure 1C–E).

The choice of the site for the experiments in the year 2020 was based on reports of outbreaks of *I. typographus* in the Apuseni Mountains (46°63′19″ N; 22°74′25″ E) (Figure 1A). In this area, after the massive felling caused by a great storm which occurred in September 2017 and which blew down about 150,000 spruce trees, bark beetle attacks were high. In the conservation area of the park, no intervention was made to clear the debris, and in the buffer zone, the debris was gradually exploited. The stand was selected in the buffer zone of the Apuseni Nature Park (managed forest), where spruce accounts for around 90% of the trees and beech accounts for 10% of the trees. The average age of trees in the stand was ~120 years; they had a consistency of 0.6 and were located at an altitude of 1170 m a.s.l. (Figure 1).

Of the trees felled during the storm and following the winter period, those that were not yet dry and had no insect infestation were chosen for the experiment. After clearing the stems of branches, they were cut into one-meter-long sections with diameters between 16 and 22 cm. The area of existing bark was calculated for each section. The sampled logs were randomly placed in the forest by hanging them upright on a dry wooden support. This was followed by labelling the four variants with six repetitions set for testing. The samples were investigated on 17 May 2020 with the onset of spring flight and sprayed on 17 May, 2 June and 16 June 2020 with the following experimental variants: variant 1 (V1) was sprayed with water; variant 2 (V2) was sprayed with *B. bassiana* 50 g/L; variant 3 (V3) was sprayed with *B. bassiana* 100 g/L; variant 4 (V4) was sprayed with *B. bassiana* 200 g/L. The amount of solution applied was 200 mL/m^2^ of bark. The concentration of *B. bassiana* powder was 2 × 109 CFU/g. Throughout the experimental period, the air temperature and humidity were recorded using the Hexo device (Figure S1 Supplementary Materials). From the middle of each log, a 30 cm-long sample of stem was collected on 30 June 2020. The resulting samples were peeled and analyzed in the laboratory. Live and dead (infected) beetles from each sample were counted following the developmental stages of larva, pupa and adults. At the time of hatching, there were no young adults (considered the next generation) in the samples. 

A new experiment was performed in the same experimental area as the previous one. Here, the aim was to observe the effects of the *B. bassiana* preparation (2 × 109 CFU (colony forming unit)/g)) on the adults of the new generations of *I. typographus*. Among the trees with broken, calcined tops, those with no insect entry were chosen for the experiment. After cleaning the stems from branches, they were sectioned into one-meter-long sections with diameters between 16 and 21 cm. The area of existing bark was calculated for each section. Logs were randomly placed in the forest by hanging vertically from a dry wood support. This was followed by labelling the four variants with six repetitions set for testing. The samples were installed on 30 June 2021, before the onset of the summer flight, and sprayed on 30 June and 14 July 2021 with the following experimental variants: variant 1 (V1) was sprayed with water; variant 2 (V2) was sprayed with *B. bassiana* 50 g/L; variant 3 (V3) was sprayed with *B. bassiana* 100 g/L; variant 4 (V4) was sprayed with *B. bassiana* 200 g/L. The amount of solution applied was 200 mL/m^2^ of bark. Other experimental conditions and procedures were as previously detailed above.

### 2.2. Data Analyses

The data of the first experiment, relating to the average length of the maternal gallery, and the data related to the mean larval/maternal galley were normally distributed; therefore, ANOVA followed by Tukey’s test were used for each experimental site (OSAL 1, 2, 3) and for the two sets of assessments separately to compare treatments.

Principal Component Analyses (PCoA were performed for each site in order to test the influence of log diameter on treatment effects (determined by the maternal gallery length), where the logs’ diameter was considered as the main component, and the adult gallery length was considered as the variable. Linear correlations were also computed between variables (log diameter and maternal gallery length). The analyses were conducted in PAST, version 4.2. Data from the second and third experiments, following parent adults, larvae, pupae and young adults’ survival rate, did not meet the assumption of normality; therefore, a Kruskal–Wallis test followed by a Wilcoxon test were used to compare survival rates. The data were also tested using PAST, version 4.2 (Oslo, Norway), with statistical differences assessed at a significance level of *p* < 0.01.

## 3. Results

**Figure 2:**
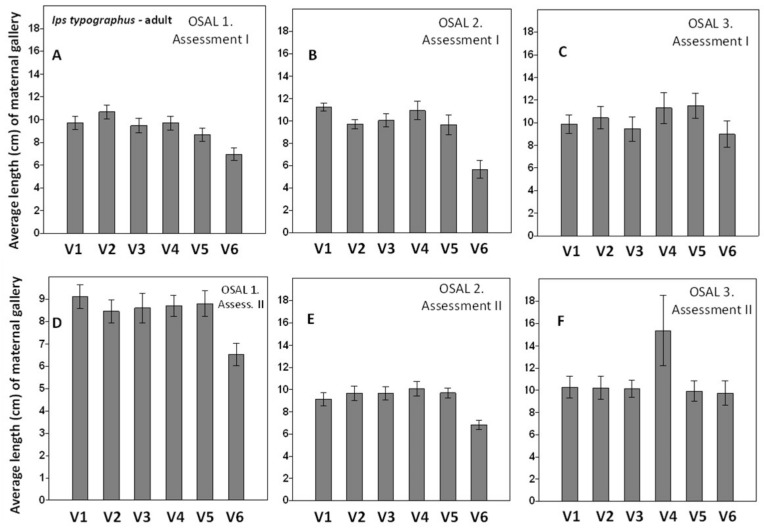
Average length (cm) of the adult *I. typographus* gallery in the first (**A**–**C**) and second (**D**–**F**) assessments: variants V1–V6 (V1, control, sprayed with water; V2, Metab 75 mL/10 L water; V3, Metab 105 mL/10 L water; V4, Metab 135 mL/10 L water; V5, Metab 165 mL/10 L water; V6, lambda-cyhalothrin 50 g/L (Karate Zeon) 50 mL/10 L water). Lines above bars represent standard errors (SE). OSAL is the official name of the Forest Management Agency in Romania.

In total, 148 logs of 50 cm in length were assessed in the first experiment, and 1480 maternal galleries and corresponding larval galleries were assessed and compared for each treatment. The effect of lambda-cyhalothrin 50 g/L was significant; smaller maternal galleries were detected after the first treatment in OSAL 1, 2. No effect between treatments at OSAL 3 was observed (Figure 2A–C, Table S1 Supplementary Materials).

The same results and a significant effect of the synthetic insecticide were observed at assessment 2 in sites (OSAL) 1 and 2, while the effect of treatment V4 (Metab 135 mL/10 L water) was less obvious than the others in site 3. No differences between the control treatment and the other treatments were detected (Figure 2D–F, Table S1 Supplementary Materials).

**Figure 3:**
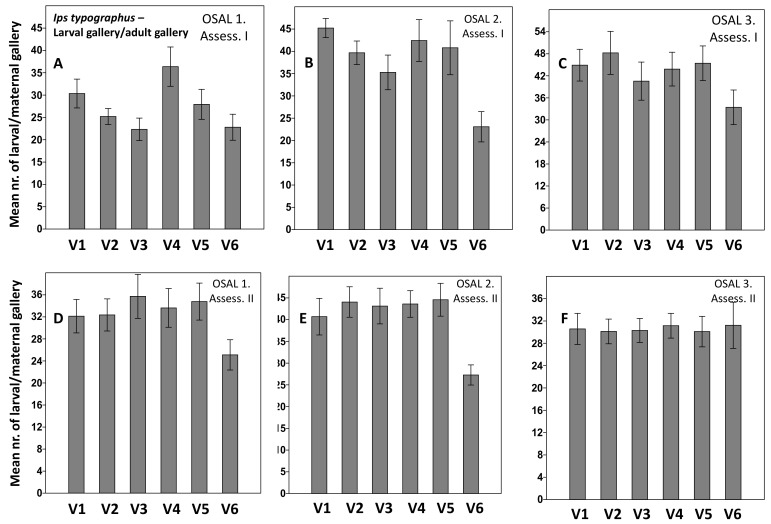
Average number of larval/adult gallery at first (**A**–**C**) and second (**D**–**F**) assessment. (V1) control, sprayed with water; variant 2 (V2) with Metab 75 mL/10 L water; variant 3 (V3) with Metab 105 mL/10 L water; variant 4 (V4) with Metab 135 mL/10 L water; variant 5 (V5) with Metab 165 mL/10 L water; variant 6 (V6) with lambda-cyhalothrin 50 g/L (Karate Zeon) 50 mL/10 L water. Lines above bars represent SE. OSAL is the official name of the Forest Management Agency in Romania.

Considering the treatment effects of the (mean larval/maternal galleries), at the first assessment, a significant influence of the lambda-cyhalothrin was observed at sites 2 and 3, while in site 1, the effects of Metab at 75 and 105 mL (V2 and V3) were significant ((Figure 3A–C, Table S2 Supplementary Materials). For the second assessment, the effect of lambda-cyhalothrin was obvious in sites 1 and 2, whereas no differences in site 3 were detected (Figure 3D–F, Table S2 Supplementary Materials).

**Figure 4:**
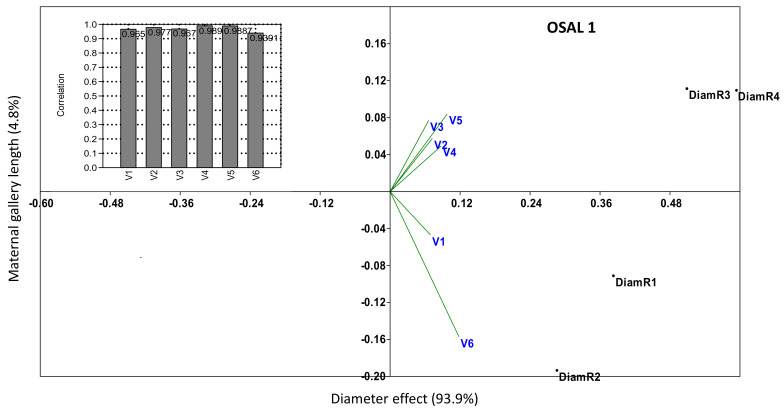
PCoA and linear correlation (bars) to test the influence of log diameter on treatments effects (determined by maternal gallery length), where the log length was considered as the main component, and the maternal gallery length was considered as the variable.

Log diameter had positive effect on adult gallery length at each treatment, log diameter determined more than 90% the presence of bark beetles, and was highly correlated with adult gallery length (Figure 4 and Figure S2B,C Supplementary Materials).

**Figure 5:**
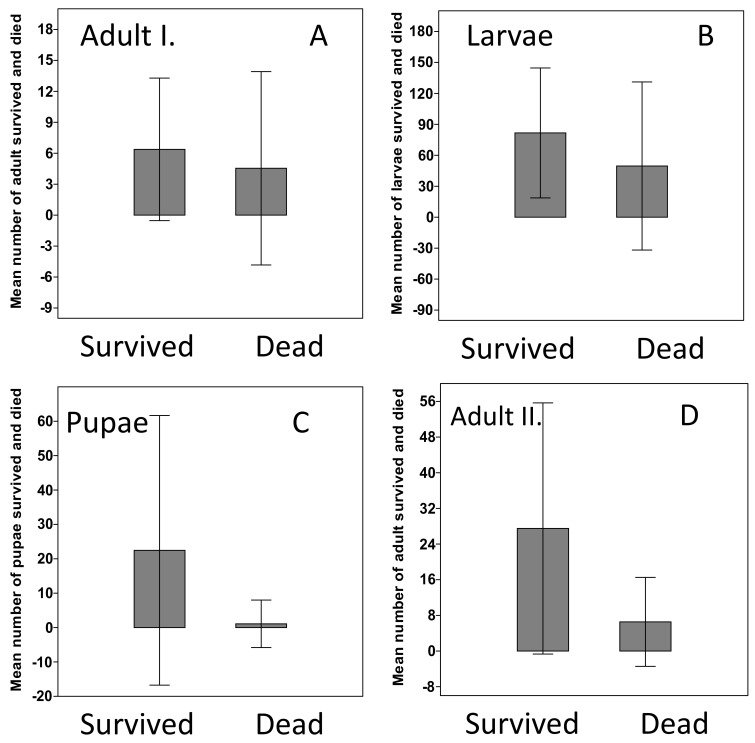
Adult, larval, pupae and next-generation adult survival rate under *B. bassiana* treatment. A Kruskal–Wallis test followed by a Wilcoxon test were used to compare survival rates. (**A**)–Adults from the start of the experiment, (**B**)–larval survival, (**C**)–Pupae survival, (**D**)–Adults from the next generations.

Altogether 4775 individual beetles were assessed to test the mortality rate. No mortality were detected at all stages when treatments were made with water (control). Adult mortality rate in treated plots changed between ~7% at the lowest, and ~77% at the highest *B. bassiana* concentration. The mortality rate increased in accordance with *B. bassiana* increasing concentrations for all developmental stages and was found to be between 28.5% and 46.8% for larvae, 7.5% and 34.3% for pupae, and 16.1% to 29.4% for young adults. By assessing the adults, larvae, pupae and the young adult (next genera-tions) survival rate, it was observed that *B. bassiana* treatment was less effective. By comparing the rate of survived and dead individuals, the survived rate was higher for larvae, pupae and for the young adults (Figure 5 and Table S3 Supplementary Materials).

## 4. Discussion

According to our results, the effect of *B. bassiana* on the maternal gallery length and the average larvae gallery number per maternal gallery was less obvious than the synthetic insecticide lambda-cyhalothrin 50 g/L. The fungal treatment effect was rather similar to the untreated variant/s. The rates of infection detected in this experiment were seemingly highly, influenced by the favorable environmental conditions for bark beetles, which was also mentioned by other authors [9]. In our experiment, the relatively low humidity during the whole experimental period probably had a positive effect on bark beetles infestation and might have reduced the effect on fungal treatment. In addition, it can be seen that log diameter was the most influential factor, influencing more than 90% of the presence of bark beetle in all research areas. Other similar studies have also reported that log diameter has the most important and positive effect on *I. typographus* infestation since, as the diameter increases, the infestation rate also increases [10,11]. In our case, highly significant correlations were detected for all sites and treatments between log diameter and *I. typographus* infection, this being higher that the effect of the fungal treatments. Beside log diameters, high temperatures also positively influenced *I. typographus* infestation and reduced the effect of *B. bassiana,* as similarly demonstrated by other authors [12,13,14]. Research in the USA has shown that bark beetle outbreaks significantly influence forest ecosystem dynamics, warming summer and winter temperatures being major drivers of beetle population outbreaks across the US [15]. 

Overall, these factors (low humidity, high log diameter and high temperature) have highly positive effects on *I. typographus* infestation capacity and can be used as broad indicators when forecasting probable tree loss. These conditions were present during the whole experimental period (Figure S1; Supplementary Materials).

The application of *B. bassiana* under field conditions had a relatively low effect on *I. typographus* stages, including adults, larvae, pupae and even next-generation young adults. Even when high product concentrations were used, the mortality rate remained relatively low. This result is in accordance with the results obtained by other studies [16]. In contrast, other similar studies demonstrated that the application of *B. bassiana* preparations in the field can cause 93% [5] to 100% [6] mortality among bark beetles in the field and 99% mortality in the laboratory [16]. Further research is necessary to test whether *B. bassiana* preparations can be applied terrestrially, aerially or in combination with pheromones in traps, as previously suggested [17]. It remains clear that the effect of *B. bassiana* preparations on bark beetle infestations in terms of possible environmental effects is minimal compared to the severe damage and hence yield loss caused by this insect species worldwide, especially in Europe and North America [18]. The economic damage caused by this insect pest is huge, so the present novel approach of using a natural biological control agent rather than toxic synthetic insecticides is hopefully timely, as it is potentially beneficial to the forestry industry as a rational control approach to combatting this major pest.

## Data Availability

Fora, Ciprian George; Boja, Nicușor; Moatăr, Mihaela; Tóth, Ferenc; Balog, Adalbert (2022): Entomopathogenic fungi *Beauveria bassiana* (Cordycipitaceae) effect on the bark beetle *Ips typographus* (L.) under field conditions. figshare. Dataset. https://doi.org/10.6084/m9.figshare.20072207.v1, accessed on 12 September 2020.

## Supplementary Materials

The following supporting information can be downloaded at: https://www.mdpi.com/article/10.3390/insects13100885/s1. **Figure S1**. Air temperature and humidity recorded during the experiment using the Hexo device. **Table S1**. Statistical values related to the average length of maternal gallery. ANOVA followed by Tukey test were used for each experimental site (OSAL 1, 2, 3) separately to compare treatments. Significant *p*-values are green marked. **Table S2**. Statistical values related to the average number of maternal/larval gallery. ANOVA followed by Tukey test were used for each experimental site (OSAL 1, 2, 3) separately to compare treatments. Significant *p*-values are green marked. **Figure S2**. Principal Component Analyses (PCoA) and linear correlation to test the influence of trunk diameter on treatments effects (determined by adult gallery length) where the trunk length was considered as main component, and adult gallery length as variables at OSAL 2 and 3. **Table S3**. The adult, larvae, pupae and next generation adult survival rate were tested with Kruskal-Wallis followed by Wilcoxon test. Significant *p*-values are green marked.
