# Supplementary material for: Effect of Entomopathogenic Fungi, Beauveria bassiana (Cordycipitaceae), on the Bark Beetle, Ips typographus (L.), under Field Conditions
Source: Insects. 2022 Sep 29;13(10):885. doi: 10.3390/insects13100885 (PMC9604305; doi:10.3390/insects13100885)
Supplement: Supplementary file 1 [file insects-13-00885-s001.zip › insects-1873529-supplementary.pdf]

Supplementary online materials

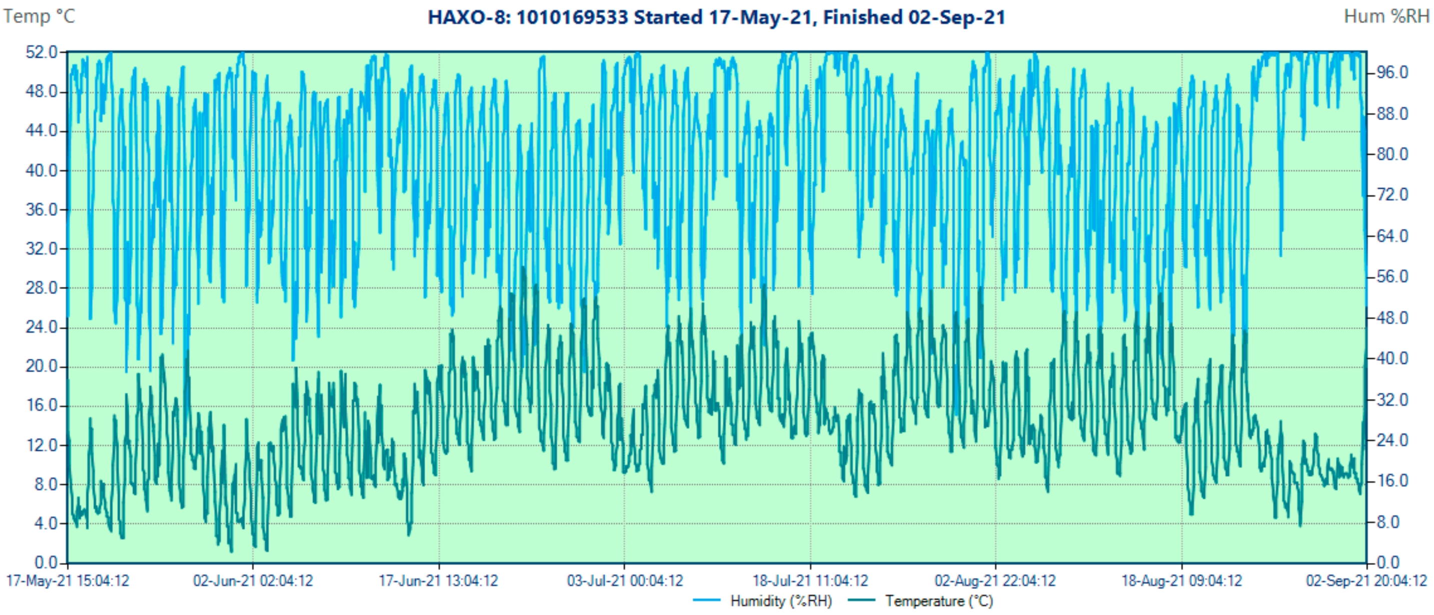

Figure S1. Air temperature and humidity recorded during the experiment using the Hexo device.

Table S1. Statistical values related to the average length of maternal gallery. ANOVA followed by Tukey test were used for each experimental site (OSAL 1, 2, 3) separately to compare treatments. Significant p values are green marked.

Fig.1A	V1	V2	V3	V4	V5	V6
V1		0.2093	0.9953	1	0.1435	0.001
V2	3.201		0.05977	0.2093	0.001	0.001
V3	0.7386	3.939		0.9953	0.3928	0.001
V4	0	3.201	0.7386		0.1435	0.001
V5	3.447	6.648	2.708	3.447		0.001
V6	9.028	12.23	8.289	9.028	5.581	

Fig.1B	V1	V2	V3	V4	V5	V6
V1		0.0223	0.1547	0.9901	0.01606	0.001
V2	4.413		0.9801	0.1221	1	0.001
V3	3.4	1.013		0.472	0.9643	0.001
V4	0.8681	3.545	2.532		0.09508	0.001
V5	4.557	0.1447	1.157	3.689		0.001
V6	16.06	11.65	12.66	15.19	11.5	

Fig.1C	V1	V2	V3	V4	V5	V6
V1		0.9832	0.9949	0.4752	0.3218	0.8701
V2	0.9745		0.8265	0.8831	0.7587	0.4549
V3	0.753	1.728		0.1867	0.1051	0.9914
V4	2.525	1.55	3.278		0.9999	0.04174
V5	2.879	1.905	3.632	0.3544		0.01944
V6	1.595	2.569	0.8416	4.12	4.474	

Fig.1D	V1	V2	V3	V4	V5	V6
V1		0.5618	0.7998	0.912	0.9735	0.001
V2	2.341		0.999	0.9883	0.9488	0.001
V3	1.8	0.5401		0.9999	0.9958	0.001
V4	1.44	0.9002	0.3601		0.9999	0.001
V5	1.08	1.26	0.7202	0.3601		0.001
V6	9.272	6.932	7.472	7.832	8.192	

Fig.1E	V1	V2	V3	V4	V5	V6
V1		0.761	0.761	0.163	0.7247	0.001
V2	1.899		1	0.9053	1	0.001
V3	1.899	0		0.9053	1	0.001
V4	3.367	1.468	1.468		0.9255	0.001
V5	1.985	0.08632	0.08632	1.381		0.001
V6	7.856	9.755	9.755	11.22	9.841	

Fig.1F	V1	V2	V3	V4	V5	V6
V1		1	1	0.001	0.9996	0.9973
V2	0.06244		1	0.001	0.9998	0.9984
V3	0.1873	0.1249		0.001	1	0.9995
V4	6.369	6.432	6.557		0.001	0.001
V5	0.4371	0.3747	0.2498	6.806		1
V6	0.6557	0.5932	0.4683	7.025	0.2186	

Table S2. Statistical values related to the average number of maternal / larval gallery. ANOVA followed by Tukey test were used for each experimental site (OSAL 1, 2, 3) separately to compare treatments. Significant p values are green marked.

Fig.2A	V1	V2	V3	V4	V5	V6
V1		0.2046	0.005306	0.08331	0.893	0.001
V2	3.216		0.8017	0.001	0.8354	0.8972
V3	5.012	1.795		0.001	0.1321	0.9999
V4	3.763	6.979	8.774		0.001	0.001
V5	1.514	1.702	3.497	5.277		0.2093
V6	4.715	1.499	0.2966	8.478	3.201	

Fig.2B	V1	V2	V3	V4	V5	V6
V1		0.393	0.001	0.9287	0.6471	0.001
V2	2.708		0.6527	0.9337	0.9989	0.001
V3	4.854	2.147		0.1341	0.3983	0.001
V4	1.366	1.342	3.488		0.9935	0.001
V5	2.159	0.5489	2.695	0.7928		0.001
V6	10.81	8.099	5.952	9.44	8.648	

Fig.2C	V1	V2	V3	V4	V5	V6
V1		0.9354	0.8252	0.9997	1	0.001
V2	1.333		0.2534	0.8145	0.9685	0.001
V3	1.731	3.064		0.9411	0.744	0.3393
V4	0.4278	1.761	1.303		0.9977	0.04012
V5	0.2089	1.124	1.94	0.6367		0.001
V6	4.567	5.9	2.835	4.139	4.776	

Fig.2D	V1	V2	V3	V4	V5	V6
V1		1	0.6536	0.9887	0.8748	0.03644
V2	0.134		0.7138	0.9947	0.9109	0.02739
V3	2.145	2.011		0.9503	0.9987	0.001
V4	0.8936	0.7596	1.251		0.9967	0.001
V5	1.579	1.445	0.566	0.6851		0.001
V6	4.185	4.319	6.33	5.079	5.764	

**Fig.2E**	**V1**	**V2**	**V3**	**V4**	**V5**	**V6**
**V1**		0.7796	0.9336	0.8671	0.6546	0.001
**V2**	1.853		0.9992	1	1	0.001
**V3**	1.342	0.5104		1	0.9932	0.001
**V4**	1.604	0.2483	0.2621		0.999	0.001
**V5**	2.143	0.2897	0.8001	0.538		0.001
**V6**	7.404	9.257	8.746	9.008	9.546	

**Fig.2F**	**V1**	**V2**	**V3**	**V4**	**V5**	**V6**
**V1**		0.9999	1	0.9997	0.9999	0.9995
**V2**	0.3157		1	0.9959	1	0.9942
**V3**	0.1929	0.1228		0.9983	1	0.9975
**V4**	0.4034	0.7191	0.5963		0.9954	1
**V5**	0.3332	0.01754	0.1403	0.7367		0.9936
**V6**	0.456	0.7717	0.649	0.05262	0.7893	

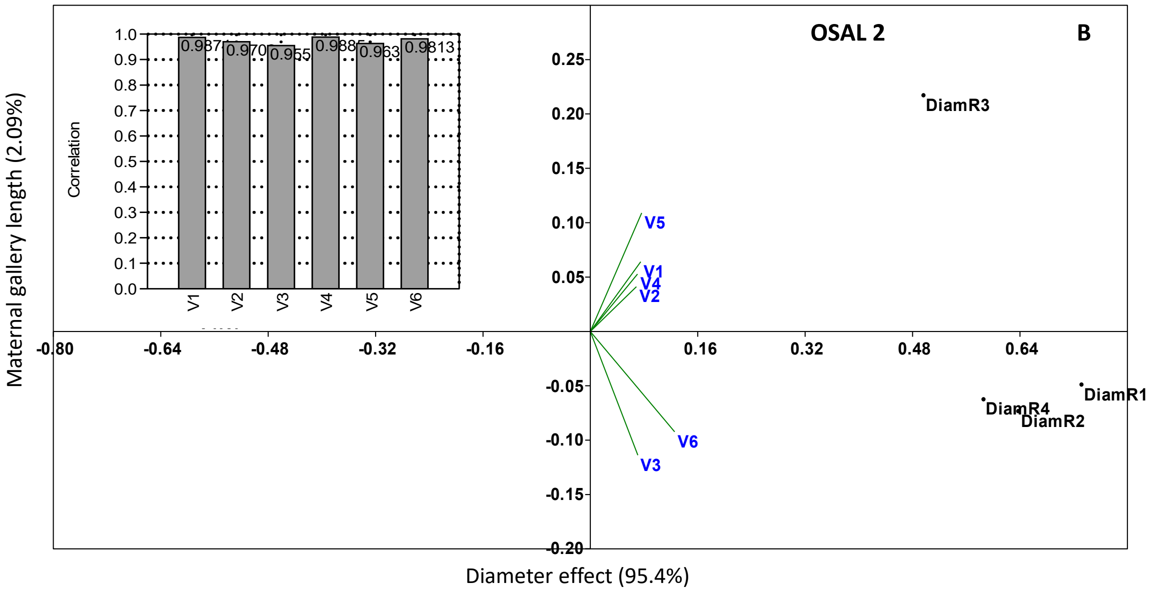

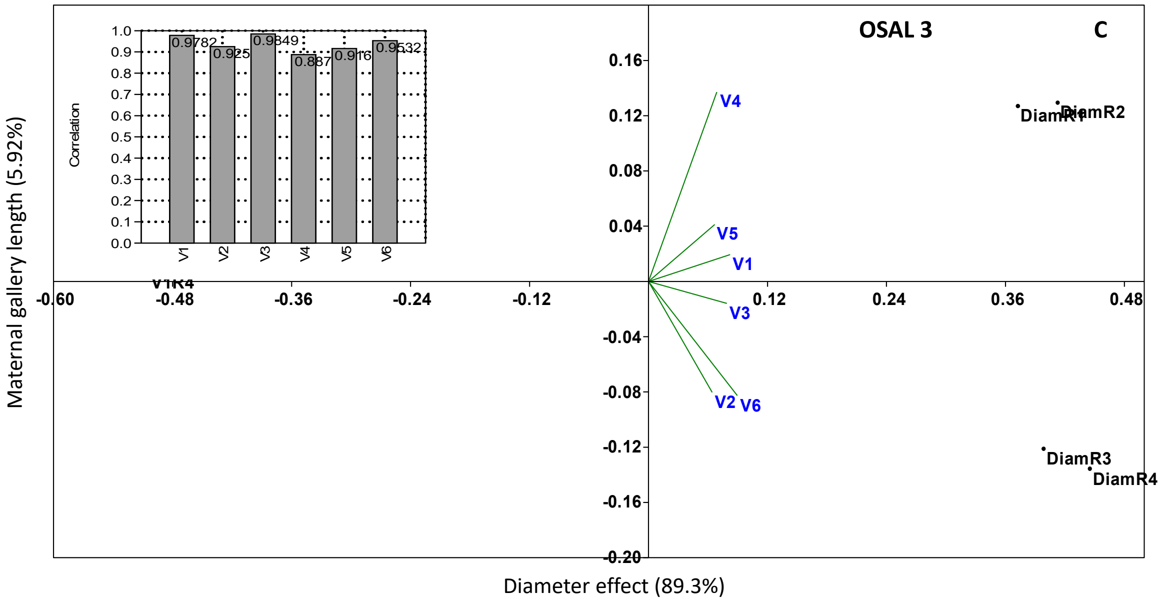

Figure S2 B, C. Principal Component Analyses (PCoA) and linear correlation to test the influence of trunk diameter on treatments effects (determined by adult gallery length) where the trunk length was considered as main component, and adult gallery length as variables at OSAL 2 and 3.

Table S3. The adult, larvae, pupae and next generation adult survival rate were tested with Kruskal-Wallis followed by Wilcoxon test. Significant p values are green marked.

**Survived adult I**		**Dead adult I**	
N:	24		
Mean:	6.375	Mean:	4.5417
Median:	6	Median:	3
WILCOXON TEST			
W:	194		
z:	1.26	p(same):	0.20782
Monte Carlo	p(same):	0.21435	
Exact	p(same):	0.21476	

**Survived larvae**		**Dead larvae**	
N:	24		
Mean:	81.667	Mean:	49.625
Median:	74.5	Median:	50.5
WILCOXON TEST			
W:	300		
z:	4.286	p(same):	0.001
Monte Carlo	p(same):	0.001	
Exact	p(same):	0.001	

**Survived pupae**		**Dear pupae**	
N:	24		
Mean:	22.458	Mean:	1.0833
Median:	18	Median:	0
WILCOXON TEST			
W:	190		
z:	3.824	p(same):	0.001
Monte Carlo	p(same):	0.001	
Exact	p(same):	0.001	

**Survived adult II**		**Dead adult II**	
N:	24		
Mean:	27.5	Mean:	6.5417
Median:	26	Median:	6.5
WILCOXON TEST			
W:	300		
z:	4.287	p(same):	0.001
Monte Carlo	p(same):	0.001	
Exact	p(same):	0.001	
